# Are Microfibers a Threat to Marine Invertebrates? A Sea Urchin Toxicity Assessment

**DOI:** 10.3390/toxics12100753

**Published:** 2024-10-17

**Authors:** Jennifer Barbosa dos Santos, Rodrigo Brasil Choueri, Francisco Eduardo Melo dos Santos, Laís Adrielle de Oliveira Santos, Letícia Fernanda da Silva, Caio Rodrigues Nobre, Milton Alexandre Cardoso, Renata de Britto Mari, Fábio Ruiz Simões, Tomas Angel Delvalls, Paloma Kachel Gusso-Choueri

**Affiliations:** 1 Laboratório de Ecotoxicologia, Universidade Santa Cecília (Unisanta), Rua Oswaldo Cruz, 266, Santos 11045-907, São Paulo, Brazil; jenniferbarbosadossantos@yahoo.com.br (J.B.d.S.); francisco.unisanta@gmail.com (F.E.M.d.S.); delvalls@unisanta.br (T.A.D.);palomakachel@unisanta.br (P.K.G.-C.); 2 MarineTox_Lab, Departamento de Ciências do Mar, Instituto do Mar, Universidade Federal de São Paulo, Campus Santos (Unifesp), Rua Carvalho de Mendonça, 144, Santos 11070-102, São Paulo, Brazil; caio.biomar@gmail.com (C.R.N.); milton.alexandre@unifesp.br (M.A.C.); fabio.simoes@unifesp.br (F.R.S.); 3 Instituto de Biociências, Campus do Litoral Paulista, Universidade Estadual Paulista (Unesp), Praça Infante Dom Henrique, s/n, Parque Bitaru, São Vicente 11330-900, São Paulo, Brazil; lais.adrielle@unesp.br (L.A.d.O.S.); leticiambiental@gmail.com (L.F.d.S.); renata.mari@unesp.br (R.d.B.M.); 4 Instituto de Ciências Ambientais, Químicas e Farmacêuticas, Unifesp, Diadema 09972-270, São Paulo, Brazil; 5 Water Challenge S.L., Avda. Papa Negro, 63, 28043 Madrid, Spain

**Keywords:** fast fashion, microplastics, anthropogenic microfibers, toxicity, embryonic development

## Abstract

The rise of “fast fashion” has driven up the production of low-cost, short-lived clothing, significantly increasing global textile fiber production and, consequently, exacerbating environmental pollution. This study investigated the ecotoxicological effects of different types of anthropogenic microfibers—cotton, polyester, and mixed fibers (50% cotton: 50% polyester)—on marine organisms, specifically sea urchin embryos. All tested fibers exhibited toxicity, with cotton fibers causing notable effects on embryonic development even at environmentally relevant concentrations. The research also simulated a scenario where microfibers were immersed in seawater for 30 days to assess changes in toxicity over time. The results showed that the toxicity of microfibers increased with both concentration and exposure duration, with polyester being the most toxic among the fibers tested. Although synthetic fibers have been the primary focus of previous research, this study highlights that natural fibers like cotton, which are often overlooked, can also be toxic due to the presence of harmful additives. These natural fibers, despite decomposing faster than synthetic ones, can persist in aquatic environments for extended periods. The findings underline the critical need for further research on both natural and synthetic microfibers to understand their environmental impact and potential threats to marine ecosystems and sea urchin populations.

## 1. Introduction

Society has been undergoing a distinct period of transition in its customs, and fashion has been following this trend. “Fast fashion”, which emerged in the 1990s, is based on the principles of producing low-cost clothing, mass production, and high sales volumes [1] with clothes that often have low durability; it is estimated that these garments may last only seven to ten wears before becoming worn out [2]. Additionally, global textile fiber production has nearly doubled in two decades, with projections indicating a quantity of approximately 150 million tons by 2030 [3]. Contamination by anthropogenic microfibers (MFs) in the environment represents a growing environmental concern on a global scale, as studies have already demonstrated their ubiquity across various environmental compartments, such as wastewater, stormwater runoff, rivers, lakes, estuaries, marine waters, and wildlife [4,5,6,7,8,9,10], with synthetic fibers being the predominant form of plastic found in marine ecosystems [11,12]. Furthermore, recent research indicates the presence of microfibers in remote regions, including the Arctic and mountain peaks [8,13].

Microfibers are any natural or artificial fibrous materials with a thread-like structure, characterized by a diameter of less than 50 μm and a length ranging from 1 μm to 5 mm [14]. These fibers have recently emerged as a pollutant of significant environmental concern due to their diverse effects on living organisms [15]. Regarding their types, fibers can be classified as natural fibers (cotton, wool), synthetic fibers (e.g., polyethylene terephthalate (polyester), polyamide (nylon)), and semi-synthetic fibers (viscose fiber, cellulose acetate, etc.) [16]. Polyester is the most widely used material in the textile market [17], and it is the primary raw material for the fast fashion industry.

It is estimated that approximately 2 million tons of microfibers are released into the ocean annually, ultimately resulting in ~1.5 million trillion microfibers in these environments [18]. Concentrations of approximately 800 microfibers/L (~0.0001 g/L) have been detected in both deep ocean environments [19] and in wastewater treatment facilities due to domestic laundry contributions [20], as well as in estuarine waters in the South Atlantic [21]. Although synthetic fiber production vastly exceeds that of natural fibers, comprising approximately 62% of global production [10], a substantial portion of the microfibers found in marine ecosystems is not of plastic origin. It is established that 88% of these microfibers are composed of natural cellulosic materials (e.g., cotton), while 12% are characterized as synthetic or semi-synthetic (e.g., polyester) [22].

In conjunction with textile industry production, one of the most predominant sources of microfibers in marine environments is associated with domestic and industrial effluents [3]. This is due to the ability of fabrics to fragment, with estimates suggesting that for every 1 kg of synthetic fabric, approximately 23,333 to 116,666 microfibers are released per washing cycle [23].

Currently, MFs are emerging as a pollutant that raises significant environmental concerns due to their diverse effects on living organisms, including alterations in reproductive processes, hormonal dysregulation, liver toxicity, and impairment of immune function [15,24]. Concerning the understanding of toxic effects on organisms, the toxicity of synthetic microfibers is, in part, better understood than the effects of natural fibers such as cotton [15], due to the biodegradability of natural fibers [25], which involves fewer efforts.

In addition to the effects of the microfibers themselves, various additives are incorporated into fibers during textile production to enhance performance, including antibacterial agents, flame retardants, dyes, plasticizers, antistatic agents, and antioxidants [26]. These substances are generally weakly bound to the fibers and can be leached into the environment [27]. Besides being a primary source of toxic agents already present in their formulation, MFs can also adsorb a wide range of contaminants from the surrounding environment, such as toxic metals and organic pollutants like PAHs, DDT, PCBs, and dioxins [28]. However, significant gaps remain in understanding the effects of MFs on marine invertebrates [29]. Conducting research that simultaneously addresses both synthetic and natural fibers is crucial for gaining a comprehensive understanding and filling knowledge gaps regarding their impacts on aquatic environments [29].

Sea urchins are key organisms in marine ecosystems, serving as an important link in trophic chains, shaping benthic rocky communities [30], and playing a vital role in material cycling and energy flow in many coastal systems [31]. The sea urchin *Echinometra lucunter* (Echinodermata: Echinoidea) (Linnaeus, 1758), used as the model organism, has significant ecological relevance [32]. The embryonic–larval development test with this species is widely used for toxicity evaluations and is well established through standard testing protocols [33,34] due to its sensitivity. On the one hand, early stages are critical for population dynamics [35]; on the other hand, this phase is the most susceptible to stress from exposure to contaminants [36]. Given their high sensitivity, previous studies have already employed the larval development of sea urchins as a bioindicator to assess the effects of microplastics [37,38,39,40,41]. However, as far as is known, the toxicity of microfibers to these organisms has not yet been evaluated, with most studies focusing on the quantification of microfibers within their tissues [20,42,43].

The objective of this study was to assess the ecotoxicological effects of different microfiber (MF) compositions on the embriolarval development of the sea urchin *Echinometra lucunter*. The specific objectives were the following: (i) to assess the toxicity of textile microfibers of cotton, polyester, and a mixture of polyester–cotton, starting with an environmentally relevant concentration and increasing to levels expected to affect the organisms (0.0001, 0.001, 0.01, and 0.1 g/L); and (ii) to simulate a scenario where the fibers remain immersed in seawater for a prolonged period to evaluate whether their toxicity changes with continuous exposure in the natural environment, considering that the toxicity may be also time-dependent. The hypothesis is that synthetic textile-derived fibers are more toxic than natural fibers and that their toxicity may change over time as they persist in the environment.

## 2. Materials and Methods

Throughout the preparation and execution of the experiments, special precautions were taken to prevent contamination by microfibers of different compositions, as well as those potentially present in the environment (air, water, etc.). These measures included the use of filtered water (GF/F glass filters, 1.2 μm pore size), even for washing materials; conducting the experiments while wearing PVC clothing; sequentially washing laboratory equipment with filtered distilled water and ethanol; and maintaining physical distance during the preparation of microfibers, different experimental solutions, as well as during the preparation of fibers and leachates.

### 2.1. Obtaining Microfibers and Preparation of Stock Solutions

The fabrics used in the study were purchased from local stores. Microfibers of cotton (100%), polyester (100%), and mixed fibers (50% cotton and 50% polyester) were manually extracted using an electric lint remover. To prevent cross-contamination, each fabric had its own dedicated remover. After extraction, the microfibers were stored and preserved in glass containers in a dark environment. To confirm the size, samples of the microfibers were imaged using an optical microscope coupled with a Motic Cam camera (Hong Kong, China). The average size was measured using the Image Pro-Plus 3.0.1 software, and the average size of the first 50 microfibers of each fabric type was calculated.

The stock solutions were prepared by first weighing different microfiber samples (100% cotton, 100% polyester, and a mixed composition of 50% cotton and 50% polyester) using an analytical balance (METTLER, TOLEDO AB204, Columbus, OH, USA). After weighing, the fibers were dispersed in a seawater at concentrations of 0.0001, 0.001, 0.01, and 0.1 g/L using a magnetic stirrer until complete dispersion was achieved. The dilution seawater (salinity of 32 and a pH of 8.2) was previously filtered through a stainless steel membrane with 48 µm pores. Immediately after, the experimental replicates were pipetted.

Two exposure scenarios were also simulated: in the first, MFs were immediately placed in seawater, and the toxicity of the experimental medium was assessed shortly thereafter; in the second scenario, MFs were immersed in seawater for 30 days at a controlled temperature (6 °C ± 2) and in the dark. After this period, the solutions were stirred without removing the microfibers, and the samples were immediately pipetted into the test tubes and the toxicity of the experimental medium was evaluated. All tests were conducted using a full-factorial design.

Water samples from the experiments were counted to estimate the average number of microfibers in each treatment, including control treatments with only water and possibly airborne microfibers. The quantification of fibers in the water samples was carried out based on Yadav and Pal [44]). After careful dispersion and agitation of the fibers, a portion of these stock solutions (1 L) was filtered using gridded ester membranes with a pore size of 0.45 µm (Química Moderna, Tetouan, Morocco). Following filtration, the membranes were placed in glass Petri dishes and incubated at 60 °C for 16 h. All fibers retained on the filters were manually counted under a stereomicroscope (BRISTOLINE, model 876453, New York, NY, USA), with the aid of a cell counter.

To determine the amount of airborne fibers present in the laboratory, open glass containers containing 1 L of filtered seawater were placed in the four corners of the room [4,45]. After the exposure period, these samples were filtered following the same protocol applied to the fiber solutions, and the fibers retained on the filters were counted. The final total of fibers in the solutions was corrected by subtracting the fibers detected in the air and in the aliquots of filtered seawater, i.e., the dilution water, which was also used in the control treatments.

### 2.2. FT-IR Characterization

Microfibers’ characterization was confirmed in the Fourier-transform infrared spectroscopy (FT-IR). In FT-IR analysis, the Spectrum Two spectrophotometer Perkin Elmer (Waltham, MA, USA), equipped with a universal attenuated total reflectance (UATR) module, was used. The equipment featured a lithium tantalate (LiTaO3) detector, a mid-infrared (MIR) source, and was controlled by the Spectrum 10 software. Prior to measurements, the ATR diamond crystal was cleaned with acetone, and a background reading was recorded. The cotton and polyester fabric samples were then compressed against the ATR diamond crystal with a force of 95 N to ensure optimal contact. The procedure, conducted in transmittance mode, involved 24 scans for each sample, covering the spectral range of 550 to 4000 cm^−1^. The analyses were performed in triplicate, and baseline correction was applied to the obtained spectra.

### 2.3. Experimental Design

This study tested various microfiber compositions (100% cotton, 100% polyester, and a mixed composition of 50% cotton and 50% polyester) and concentrations of microfibers (0.0001, 0.001, 0.01, and 0.1 g/L). The values of items/MF/L for each treatment are detailed in Table 1.

For the embryonic–larval development tests (subchronic toxicity assay), adult specimens of *Echinometra lucunter* were collected by free diving at Ilha das Palmas (Guarujá, São Paulo, Brazil) in November 2023, during the spring. The specimens were transported to the laboratory in a portable cooler and kept in tanks with natural seawater under controlled conditions (dissolved oxygen, 5.5 ± 0.5 mg/L; salinity 34 ± 2; pH 8.3 ± 0.2; 16/8 h light/dark cycle). The test followed standard procedures (NBR 15350) [33]. After a two-day acclimation period, the organisms were rinsed with dilution water to remove any debris from the body surface and stimulated to release their gametes through the application of a 5 mL injection of 0.5 M KCl in the perioral region (2.5 mL at each point). The animals were then gently agitated to allow the KCl to spread throughout their coelomic cavity. Male and female gametes were separately collected from 3 individuals of each sex (the gametes differ in color, with oocytes being orange and sperm being whitish).

The sperm was collected directly from the gonopores using a micropipette and stored in a beaker on ice until fertilization. Oocytes were collected in beakers containing seawater, with the females positioned aboral side down. After the release of the oocytes, the content was filtered through a 350 μm mesh and combined into a single beaker containing the oocytes from the 3 females, with seawater added to a final volume of 600 mL. The oocytes were allowed to settle, and the supernatant was discarded. This process was repeated three times.

A sperm suspension was prepared by mixing sperm with dilution seawater to dissolve any clumps. This suspension was then added to the oocyte suspension, and the mixture was gently stirred for 5 min to allow fertilization, resulting in the formation of a fertilized egg suspension. To confirm fertilization, 1 mL of the egg suspension was diluted in 100 mL of dilution seawater. From this mixture, 1 mL was placed in a Sedgwick–Rafter chamber for egg counting under a light microscope. The required volume of fertilized egg suspension per replicate was calculated and added to the test solutions 2 h after fertilization. The density of units per volume was estimated to allow for the addition of sufficient organisms to each test chamber, achieving an approximate density of 400 eggs/mL.

The assays were considered valid when at least 80% of the control organisms reached the normal pluteus stage, characterized by arms of equal or greater length than the larval body. The larvae were counted in a Sedgwick–Rafter chamber under a light microscope.

Tests were conducted in glass test tubes containing 10 mL of experimental medium, and constant conditions were maintained throughout the experiment (16:8 h light/dark cycle; temperature 28 ± 2 °C; salinity 34). After a 36 h incubation period under static conditions, larvae were fixed in buffered formaldehyde (10%), and the percentage of normal larvae was determined under an optical microscope (400× magnification) with the aid of a Sedgwick–Rafter chamber. The test was considered acceptable if more than 80% normal development was observed in negative controls. Larvae with delayed development compared to the control were also considered affected.

For each treatment, four replicates were used, and the physicochemical parameters (pH, salinity, dissolved oxygen, and temperature) were checked at the beginning and end of the assay. Salinity was measured with a refractometer (Instrutherm, model: RTS-101ATC, São Paulo, Brazil), pH was analyzed using a pH meter equipped with a glass electrode specific for pH (Digimed, model: DM-23-DC, São Paulo, Brazil), and dissolved oxygen was measured with a dissolved oxygen meter (WTE, model: Oxi 315i, Essen, Germany). A parallel sensitivity test with a reference toxic substance (ZnSO_4_·7H_2_O) [46] was conducted, and the results were compared with a control chart maintained by the laboratory.

### 2.4. Data Analyses

The significance of differences between treatments was assessed using a Univariate Permutational Analysis of Variance (PERMANOVA) [47] with a two-factor design to analyze the effect of fixed factors: the condition (2 levels: freshly added MFs and aged MFs) and concentration (4 levels: 0.0001, 0.001, 0.01, 0.1 g/L) for each type of MF and the mixture. Another PERMANOVA was conducted to evaluate differences in toxicity between MF types, considering three fixed factors: the MF type (3 levels: polyester, cotton, and mixed), as well as the condition and concentration as previously described. When the main test yielded significant results, a post hoc “paired multiple comparisons test” was applied to identify which levels differed from each other. The PERMANOVA tests were conducted on Euclidean distance matrices, and residuals were permuted using unrestricted permutation of raw data. Variance homogeneity was analyzed using PERMDISP. The significance level was set at α = 0.05 for all tests.

## 3. Results

### 3.1. Size and Composition of Microfibers

**Figure 1:**
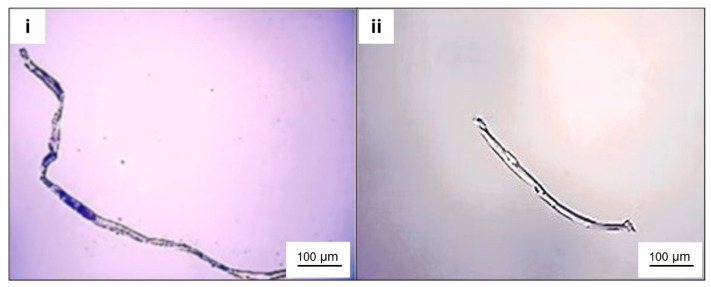
Photomicrographs of cotton microfibers (**i**) and polyester microfibers (**ii**).

The microscopic measurement of cotton microfibers yielded an average length of 490 ± 25 μm, an average diameter of 14 ± 2 μm, and a total area of 5114 ± 46 μm^2^ (Figure 1i). Polyester microfibers showed an average length of 424 ± 25 μm, an average diameter of 17 ± 3 μm, and a total area of 7040.95 ± 46 μm^2^ (Figure 1ii). These values are within the ranges described by Desforges et al. [48].

**Figure 2:**
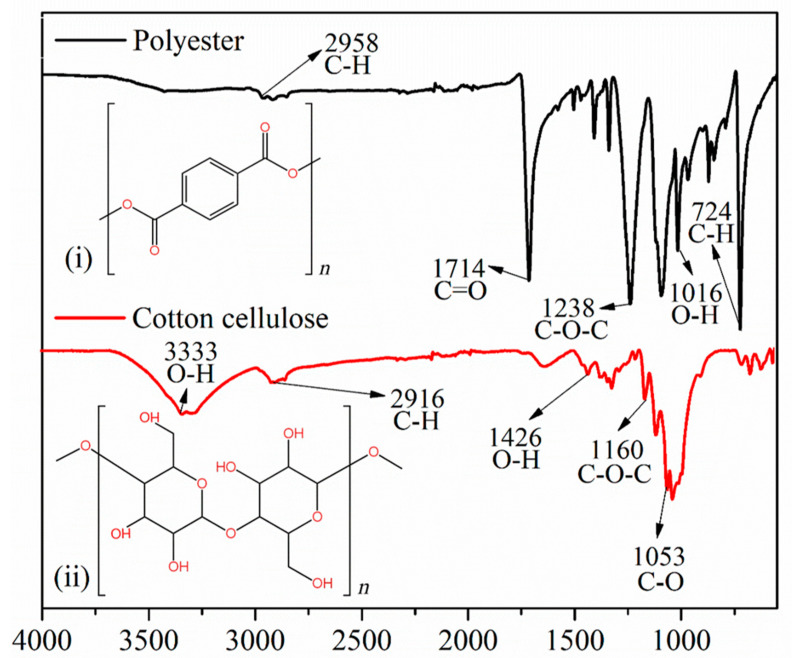
FT-IR (UATR) spectra of polyester and cotton samples covering the spectral range from 550 to 4000 cm^−1^. (**i**) Chemical structure of polyester. (**ii**) Chemical structure of cotton cellulose.

Figure 2 shows representative spectra of the polyester and cotton samples. Polyester, a synthetic fiber, primarily consists of polymer chains made from repetitive units of diacid esters and diols [49]. These polyesters are commonly derived from polymers such as polyethylene terephthalate (PET). Thus, in Figure 2i, the chemical structure of polyester is illustrated [50], which facilitates the identification of the bands highlighted in the spectrum. Similarly, Figure 2ii presents the chemical structure of cellulose [51], the primary component of cotton.

In the polyester spectrum in Figure 2i, characteristic bands of fibers manufactured from PET are observed. For example, weak C-H stretching (2958 cm^−1^), the presence of the carboxylic group in the molecule (1714 cm^−1^), O-H out-of-plane bending in the terminal carboxylic groups (1016 cm^−1^) [52], the presence of an ester single bond C-O-C (1238 cm^−1^) [53], and C-H bending (724 cm^−1^) [54] are noted. Meanwhile, in the spectrum of cotton cellulose, vibration stretching in the O-H bond (3333 cm^−1^) [55] is evident, as well as stretching in the C-H bond (2916 cm^−1^), symmetric bending in O-H (1429 cm^−1^), and C-O-C and C-O stretching at 1160 cm^−1^ and 1053 cm^−1^, respectively [56]. Other bands present at 1315 cm^−1^, 1018 cm^−1^, and 1029 cm^−1^ also characterize the FT-IR spectra of cellulose [52,57,58].

### 3.2. Toxicity of New and Aged MFs to Echinometra lucunter

The EC50 for sea urchins was 0.34 mg/L ZnSO_4_, indicating that the organism’s sensitivity was within the acceptable range established by the control chart maintained in the laboratory (0.15–0.58 mg/L ZnSO_4_).

The results of the statistical analyses of the toxicity tests for *E. lucunter* are presented in Table 2. Initially, each type of microfiber (cotton, polyester, or mixed) was tested in a two-factorial design considering its condition (new or aged) and different concentrations. Subsequently, a three-factor PERMANOVA was conducted, incorporating all types of fibers, their respective conditions, and concentrations.

**Figure 3:**
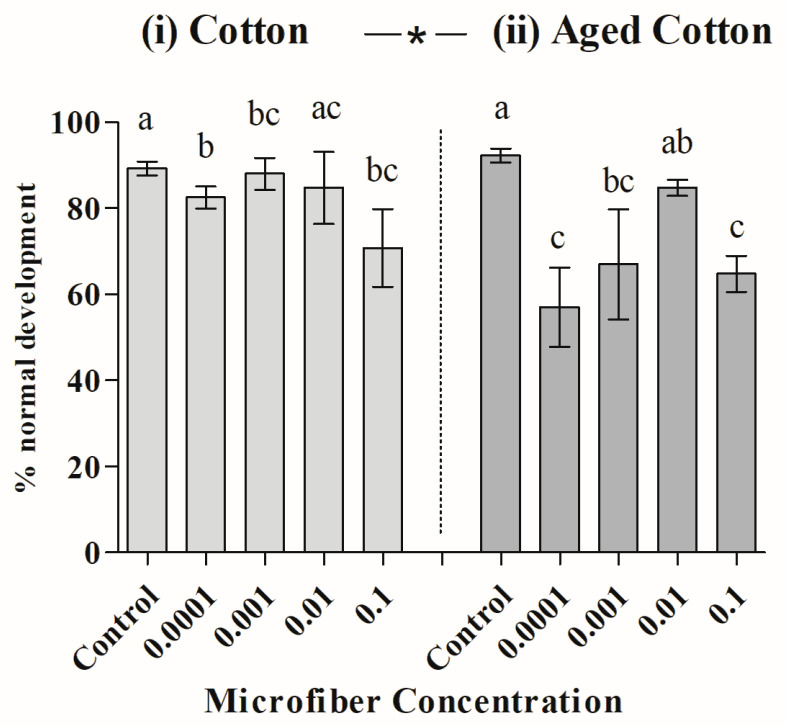
Mean ± standard deviation (SD) of normal sea urchin embryo–larval development exposed to different concentrations (g) of microfibers of (**i**) freshly added cotton and (**ii**) aged cotton. The asterisks indicate significant differences between new and aged microfibers. Different letters represent significant differences between concentrations.

Figure 3 shows the toxicity test results using cotton fiber at different test concentrations. The results indicate that both factors (condition and concentration) had a significant effect, although the interaction was not significant (PERMANOVA; *Pseudo-F(4,30)* = 1.88; *p* = 0.114). Toxicity increased (i.e., a reduction in the percentage of normally developed larvae) with aged fibers. Additionally, higher toxicity was observed at concentrations of 0.0001, 0.001, and 0.1 g/L compared to the control treatments (Table 2), with these differences being more pronounced for aged cotton. PERMDISP analysis revealed that the dispersions between the levels of these MFs are heterogeneous (Levene’s test; *F(1,38)* = 11.57, *p(perm)* = 0.001).

**Figure 4:**
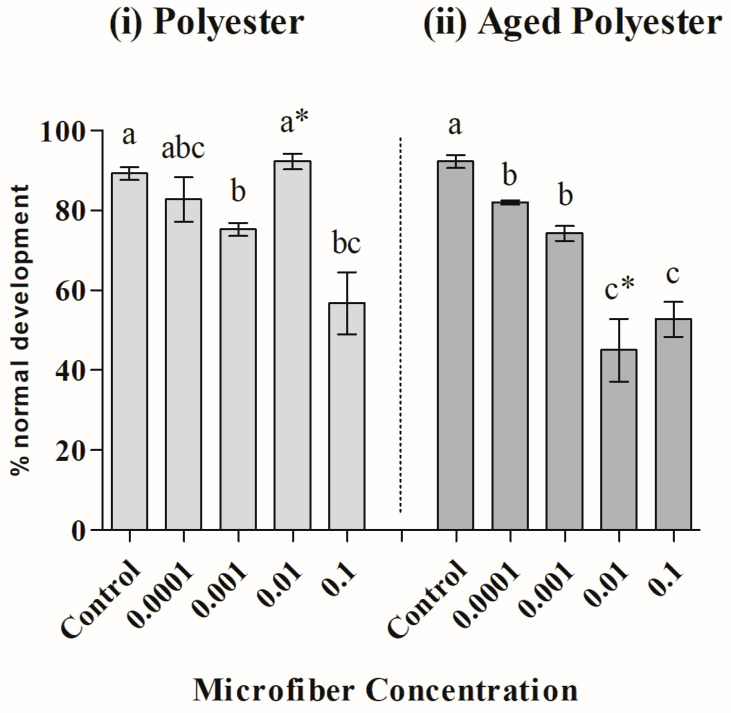
Mean ± standard deviation (SD) of normal sea urchin embryo–larval development exposed to different concentrations (g) of microfibers of (**i**) freshly added polyester and (**ii**) aged polyester. The asterisks indicate significant differences between new and aged microfibers. Different letters represent significant differences between concentrations.

When analyzing the results for polyester microfibers (Figure 4), PERMDISP analysis indicated that the variations between treatments are also heterogeneous (Levene’s test; *F(9,30)* = 3.49, *p(perm)* = 0.015). PERMANOVA showed a significant interaction between the tested variables (PERMANOVA; *Pseudo-F(4,30)* = 9.83; *p* = 0.01), and paired post hoc tests revealed that aged microfibers were more toxic than newly added ones, but only at the concentration of 0.01 g/L. The differences within each condition (newly added or aged MFs) showed that newly added fibers were different and more toxic than the control at concentrations of 0.001 and 0.1 g/L. In contrast, aged fibers were significantly more toxic than the control at all concentrations tested (Table 2).

**Figure 5:**
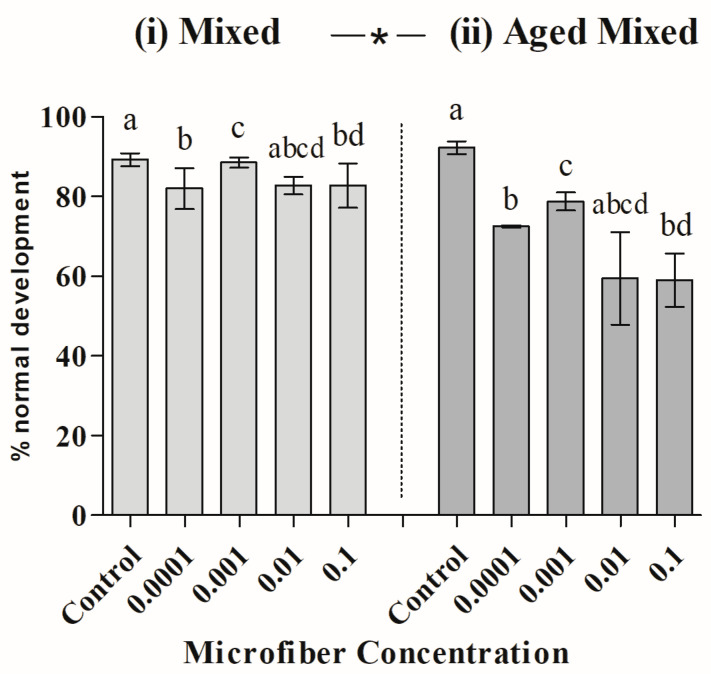
Mean ± standard deviation (SD) of normal sea urchin embryo–larval development exposed to different concentrations (g) of microfibers of (**i**) freshly added mixed microfibers and (**ii**) aged mixed microfibers. The asterisks indicate significant differences between new and aged microfibers. Different letters represent significant differences between concentrations.

The mixed composition microfibers (50% cotton and 50% polyester) also exhibited heterogeneous variances for the ‘Condition’ factor (Levene’s test for ‘Condition’; *F(1,38)* = 4.91, *p(perm)* = 0.027), but not for the ‘Concentration’ factor (Levene’s test for ‘Concentration’; *F(4,35)* = 1.82, *p(perm)* = 0.11) according to PERMDISP analysis. PERMANOVA demonstrated significant differences in toxicity related to both conditions (newly added or aged), regardless of the tested concentration (PERMANOVA; *Pseudo-F(1,30)* = 9.20; *p* = 0.002), and among different concentrations, regardless of whether the mixed MFs were newly added or aged (PERMANOVA; *Pseudo-F(4,30)* = 3.58; *p* = 0.012) (Table 2). Paired post hoc tests showed that aged MFs are more toxic than newly added MFs. Regarding the effects of MF concentration, the results indicated that all concentrations are significantly more toxic than the control, with toxicity tending to increase with higher concentrations of MFs. Although the interaction between factors was not significant, this effect is more pronounced for aged MFs (Figure 5).

The three-factor PERMANOVA analysis testing the effects of condition, concentration, and type of microfibers (MFs) on the embryonic–larval development of *E. lucunter* revealed a significant interaction among these three factors (PERMANOVA; *Pseudo-F(6,72)* = 3.61; *p* = 0.002) (Table 2). This indicates that the difference in toxicity between the types (or compositions) of MFs is dependent on both the age condition and the concentration of these particles. Paired tests highlight that for newly added fibers at the environmentally relevant concentration (0.0001 g/L), there are no significant differences in toxicity among the types of MFs, all being non-toxic. At the higher concentration (0.1 g/L), all MFs are equally toxic. For newly added fibers, at intermediate concentrations, polyester alone is more toxic than cotton and mixed fibers at 0.001 g/L, while at 0.01 g/L, polyester is more toxic only compared to the mixed microfibers.

For microfibers that remained in the water for a longer period, it is notable that polyester MFs, at the environmentally relevant concentration (0.0001 g/L), are significantly less toxic than other compositions. At the immediately higher concentration tested (0.001 g/L), polyester exhibits greater toxicity than cotton, while at 0.01 and 0.1 g/L, all types of microfibers are equally toxic to the organisms.

## 4. Discussion

The results of this study demonstrated that all three types of anthropogenic microfibers used (cotton, polyester, and a polyester–cotton mixture) can be toxic to organisms in both current and likely future scenarios. It was also observed that cotton fibers, despite being derived from natural sources, can affect the embryonic–larval development of *E. lucunter* even at environmentally relevant concentrations (0.0001 g/L). Most studies on the toxicity of anthropogenic microfibers in aquatic organisms have predominantly used synthetic fibers, e.g., [50,51,52,53,54]. Studies on natural fibers are still limited [59]. However, scientific knowledge needs to advance regarding the toxicity of microfibers of all compositions, as organisms are exposed to both natural and synthetic fibers, e.g., [60,61].

One of the reasons for the lack of research on natural microfibers, such as those derived from cotton, is partly attributed to their faster decomposition rates compared to synthetic fibers, which leads to reduced persistence in environmental samples [62,63]. However, it should be noted that these materials can still remain in aquatic environments for periods ranging from months to decades, depending on the type of fiber and the environmental factors involved [4,62,63]. Human modifications, such as the use of dyes and special treatments, can also increase the degradation time of these fibers in the environment [64]. These characteristics and their continuous entry into ecosystems may lead to them being considered “pseudo-persistent”.

The observed toxicity of cotton microfibers in this study is believed to be due to the fact that, despite being derived from natural materials, natural microfibers often contain a range of chemical additives, dyes, and finishing agents added during textile production. These chemical additives can include toxic compounds such as bisphenols, azo dyes, perfluorinated alkyl compounds (PFASs), and formaldehyde. In contrast, plastic microfibers may contain an even greater number of additives [63,64,65].

Although cotton microfibers showed significant toxicity, synthetic microfibers, such as polyester, and the polyester–cotton mixture exhibited even greater toxicity. Previous studies have demonstrated that natural microfibers can be relatively less toxic compared to synthetic microfibers [29,66]. However, they have still shown adverse effects on growth and behavior in mysid shrimp (*Americamysis bahia*) and fish (*Menidia beryllina*) [66], as well as on mussels (*Mytilus* spp.) [29]. In contrast, for brine shrimp (*Artemia franciscana*), the toxicity results were similar for commonly used synthetic microfibers (polypropylene and polyethylene terephthalate) and a natural fiber (lyocell) [59].

This study found that as the concentration of microfibers in the water increases, the toxic response also increases, particularly for polyester. The literature also highlights the effects of microfibers derived from plastic materials, such as impacts on embryonic–larval development, reduced food consumption rates, growth and reproduction, increased adult mortality, decreased filtration rates, as well as oxidative stress and negative embryonic development [40,67,68,69].

In the present study, two exposure scenarios were tested for *E. lucunter* embryos: the first scenario involved newly introduced microfibers, and the second scenario involved microfibers that had been immersed in seawater for 30 days. The results demonstrated a trend of increasing toxicity as the microfibers remained in the environment longer, a phenomenon observed for all tested compositions (cotton, polyester, and mixed). This effect is believed to be due to the leaching of chemicals from the microfibers. Indeed, recent studies suggest that leached additives are a primary cause of toxicity to aquatic organisms [40,70]. The impact of leached plastics on sea urchins has been studied for various materials [37,39,40,71,72]. However, there is a gap in understanding the effects of leachates from textile microfibers on aquatic organisms [73]. Furthermore, it is necessary to expand knowledge on which potentially toxic chemicals are present in textiles and how they leach into aqueous environments under conditions that simulate environmental degradation processes [74]. These chemical additives tend not to be covalently bonded to the polymer matrix, making them more prone to leaching into aquatic environments [64,74,75].

In addition to leached substances, it cannot be ruled out that the microfibers themselves might have had direct impacts on the health of aquatic organisms. Small particles fall within the same size range as microalgae species ingested by sea urchin larvae (i.e., <10 μm), making them easily mistaken for food by the larvae. Indeed, a previous study demonstrated the ingestion of plastic microparticles by sea urchin larvae [76].

The present study evaluated toxicity at endpoints such as reproduction even at environmentally relevant concentrations of microfibers, such as those reported for coastal waters and other aquatic environments [19,20,21]. The observed toxicity in this study is therefore of immediate concern, as likely future scenarios for microfibers in coastal waters suggest that as global rates of textile production and consumption increase, and if management measures are not implemented, microfiber concentrations in the ocean are expected to rise correspondingly.

These scenarios are particularly concerning in developing countries, where wastewater treatment facilities often lack the technology to remove anthropogenic plastic microparticles [77]. Additionally, due to challenges in measuring and identifying fibers in environmental samples, reported levels of anthropogenic microfibers in marine environments may still be underestimated [4].

The larval stage is widely recognized as the most sensitive period in the life cycle of sea urchins to the effects of contaminants [78]. Impacts during this developmental stage, such as reduced larval survival rates and settlement success, can compromise the long-term viability of adult populations [79,80]. However, in addition to the pressures on this life stage, previous studies have also demonstrated the ability of adult individuals to assimilate microfibers, which could potentially affect the physiology of these organisms and further pressure their populations. For instance, in the Adriatic Sea, adult sea urchins contained an average of 4 microfiber items per individual [5]; in the Mediterranean Sea, the average was 2.6 items per individual [81], while in the Aegean Sea (Greece), an average of 1.95 ± 1.70 microfibers per gram of soft tissue was detected [82]. In the coastal area of northern China, the presence of microfibers in the digestive system of four different sea urchin species has also been documented, with values ranging from 2.20 ± 1.50 to 10.04 ± 8.46 items per individual [43].

It is believed that microfibers are internalized by sea urchins through the peristomial membrane and then transferred to the fluids and gonads [43]. The same authors revealed the presence of both natural and synthetic microfibers, attributing this assimilation to the fact that these materials are denser than seawater (cotton: 1.5 g/cm^3^; polyester: 1.4 g/cm^3^), suggesting that these fibers tend to settle on the seafloor due to gravity.

Sea urchins play a key role in the food chain as prey for carnivorous fish and crustaceans, and any deleterious effects on their life cycle (as reported in this study), survival, or growth may result in changes at other levels of the ecosystem [83]. Sea urchins also provide a variety of highly relevant ecosystem services, such as acting as community modifiers for algae, maintaining biodiversity in marine ecosystems, nutrient cycling, cycling organic matter into forms that can be utilized by other marine organisms, and offering commercial value as a source of food and income for fishing communities [84].

Considering the global changes the planet is currently experiencing, with the acceleration of the “fast fashion” clothing consumption model, it is assumed that textile microfibers, if no management actions are developed to prevent their entry into the ocean, could significantly affect sea urchin populations and, consequently, the benthic ecosystems that depend on them [85,86]. The use of new disruptive technologies could help to avoid the entrance of these substances from the industrial textile processes in the aquatic environment, namely the ocean, however it is a pity that few of them are usually running at industrial levels [87].

## 5. Conclusions

The results of the present study demonstrated that all three types of anthropogenic microfibers tested (cotton, polyester, and a polyester–cotton blend) can be toxic to aquatic organisms, both in current scenarios and in likely future scenarios. It was observed that cotton microfibers, although derived from natural sources, affected the embryonic–larval development of *E. lucunter* even at environmentally relevant concentrations (0.0001 g/L). It is worth exploring in future research whether the toxicity of cotton MFs is due to the presence of chemical additives, dyes, and finishing agents used in cotton cultivation or textile production, many of which are toxic. Synthetic microfibers, such as polyester, exhibited even greater toxicity than cotton MFs.

The study also revealed that increases in microfiber concentrations in water resulted in rising toxic responses, particularly for polyester. Toxicity increased as the microfibers remained in the environment for longer periods, which may be due to the leaching of chemical substances from the microfibers. The toxicity of microfibers is an immediate concern, especially considering that global rates of textile production and consumption are on the rise. Future scenarios indicate that, without management measures, the concentrations of microfibers in the oceans are expected to increase.

The larval stage of sea urchins is recognized as the most sensitive period to the effects of contaminants, and exposure to microfibers may compromise the long-term viability of adult populations. Given the acceleration of “fast fashion” clothing consumption, it is essential to develop management actions to prevent the entry of microfibers into the oceans, as they may significantly affect sea urchin populations and the benthic ecosystems that depend on them.

This study presents some limitations that should be considered. Firstly, the experimental conditions were conducted in a controlled environment, which may not fully reflect the complexity and variability of natural aquatic ecosystems. In these environments, factors such as temperature, salinity, and the presence of other pollutants can significantly influence the toxicity of microfibers. Furthermore, although different types of microfibers were tested, the diversity of materials used in the textile industry is extensive, and microfibers from other sources and compositions were not included in this research. It is also important to highlight that studying sublethal effects at lower levels of biological organization, such as cells and tissues, could provide a more accurate prediction of the impacts of these materials and contribute to more effective management of contaminants. These limitations emphasize the need for future investigations that seek to elucidate more comprehensively the impacts of microfibers on aquatic ecosystems.

## Figures and Tables

**Table 1:** Number of microfiber items per liter in samples.

Microfiber	Concentration (g/L)	Microfiber Itens/L
Cotton	0.0001	835
Cotton	0.001	6206
Cotton	0.01	66,220
Cotton	0.1	238,546
Polyester	0.0001	884
Polyester	0.001	8523
Polyester	0.01	59,336
Polyester	0.1	268,884
Mixed fibers	0.0001	916
Mixed fibers	0.001	5940
Mixed fibers	0.01	68,530
Mixed fibers	0.1	284,900
Control treatment	--	37
Airbone	--	60

**Table 2:** PERMANOVA results based on the effect of different microfiber combinations on the larval development of Echinodermata and multivariate analysis. df = degrees of freedom; MS = mean squares; P(perm) = permutational *p*-value; Unique perms = number of unique permutations. Significant *p*-values are marked in bold. Conditions: new and aged; concentrations: 0.0001, 0.001, 0.01, 0.1 g/L; types: cotton, polyester, or mixed. The associations that showed statistical significance are highlighted in bold.

Main Test	df	MS	Pseudo-F	P (perm)
**Cotton**				
**Condition**	**1**	**1500.6**	**10.099**	**0.006**
**Concentration**	**4**	**627.55**	**4.223**	**0.007**
Condition vs. Concentration	4	279.87	1.884	0.114
**Polyester**				
**Condition**	**1**	**1210**	**13.378**	**0.003**
**Concentration**	**4**	**1224.1**	**13.533**	**0.001**
**Condition vs. Concentration**	**4**	**889.12**	**9.83**	**0.001**
**Mixed**				
**Condition**	**1**	**1276.9**	**9.203**	**0.002**
**Concentration**	**4**	**495.96**	**3.575**	**0.012**
Condition vs. Concentration	4	196.09	1.4132	0.26
**Three-way PERMANOVA with all treatments**				
**Type of MF**	**1**	**5922**	**42.539**	**0.001**
Condition	2	239.95	1.666	0.185
**Concentration**	**3**	**473.74**	**3.403**	**0.025**
**Type × Condition × Concentration**	**6**	**502.04**	**3.606**	**0.002**

## Data Availability

Dataset available on request from the authors. The raw data supporting the conclusions of this article will be made available by the authors on request.
